# Hepatic arterial infusion chemotherapy plus sintilimab as conversion therapy for locally advanced hepatocellular carcinoma: a single-center phase II trial

**DOI:** 10.1097/JS9.0000000000004692

**Published:** 2026-01-13

**Authors:** Yangxun Pan, Xiaohui Wang, Chuan Peng, Jing Zhao, Shixun Lu, Chao Zhang, Juncheng Wang, Yizhen Fu, Zhongguo Zhou, Minshan Chen, Yaojun Zhang, Li Xu

**Affiliations:** a Department of Liver Surgery, Sun Yat-sen University Cancer Center, Guangzhou, China; b Collaborative Innovation Center for Cancer Medicine, State Key Laboratory of Oncology in South China, Guangzhou, China; c Guangdong Provincial Clinical Research Center for Cancer, Guangzhou, China; d Department of Hepatobiliary Surgery, Hunan Provincial People’s Hospital, The First Affiliated Hospital of Hunan Normal University, Changsha, Hunan, China; e Department of Ultrasound, Sun Yat-sen University Cancer Center, Guangzhou, China; f Department of Radiology, Sun Yat-sen University Cancer Center, Guangzhou, China; g Department of Pathology, Sun Yat-sen University Cancer Center, Guangzhou, China

**Keywords:** clinical trial, conversion therapy, hepatic arterial infusion chemotherapy, hepatocellular carcinoma, programmed cell death-1 inhibitor

## Abstract

**Introduction:**

Patients with locally advanced hepatocellular carcinoma (aHCC) who have macroscopic vascular invasion typically have a poor prognosis. As the understanding of interventional therapy evolves, the conversion therapy strategy based on interventional approaches can significantly enhance objective response and raise the chance of resection for these patients. While the long-term outcomes of the conversion-to-surgery strategy remain unclear. In this article, we report the results of a phase II clinical trial that focused on the survival benefits and safety of conversion-to-surgery therapy, utilizing hepatic arterial infusion chemotherapy of infusion fluorouracil, leucovorin, and oxaliplatin (FOLFOX-HAIC) combined with sintilimab for locally aHCC.

**Methods:**

This open-label, single-center, two-cohort phase II study recruited a total of 40 patients who had initially unresectable HCC within SYSUCC criteria between March 2019 and July 2020. Patients received FOLFOX-HAIC, combined with intravenous sintilimab (cohort A, *N* = 30) or not (cohort B, *N* = 10) every 3 weeks for up to eight courses. Patients who achieved tumor shrinkage and were eligible for surgery received radical resection or ablation, then received sintilimab monotherapy up to 16 doses in total (cohort A) or supportive care (cohort B). The primary endpoint was progression-free survival (PFS) assessed with the Response Evaluation Criteria in Solid Tumors version 1.1.

**Results:**

Of the total 40 subjects, the median age was 50 years (range, 29–70 years). With a median follow-up of 52.9 months (range, 6.6–68.6 months). The median PFS was 19.8 months [95% confidence interval (95% CI), 8.2 months-not reached (NR)] in cohort A, and 17.6 months (95% CI, 4.13 months-NR) in cohort B. The conversion rate was beyond 70% in both cohorts. Of the 26 patients (19 in cohort A and 7 in cohort B) who received liver resection, 4 of cohort A (21%) and 1 of cohort B (14%) achieved pathological complete response. The 5-year overall survival rate of the whole study population was 56.3% (95% CI, 43–74%).

**Conclusions:**

Hepatic arterial infusion chemotherapy plus sintilimab exhibited impressive outcomes and safety as a conversion therapy for locally aHCC.

## Introduction

Primary liver cancer is the third leading cause of cancer mortality globally, with hepatocellular carcinoma (HCC) accounting for 75–85% of primary liver cancer cases. China contributes to nearly half of HCC cases globally^[1,2]^. Moreover, nearly half of Chinese HCC patients are diagnosed with advanced-stage disease at their initial presentation, facing a poor median survival of less than 5 months when receiving only supportive care^[3,4]^.

Recently, an increasing number of initially unresectable HCC patients have gained opportunities for surgical treatment following locoregional therapy, systemic therapy, either individually or in conjunction, which has gathered increasing consensus and attention for the conception of “conversion strategy”^[5–7]^. According to the current consensus, conversion therapy for HCC aims to eliminate the factors of “unresectability” from either technical or oncological aspects^[5,8]^. However, the long-term survival benefit of the conversion-to-surgery strategy for unresectable HCC is not well elaborated.

In the previous retrospective studies, we found that locally advanced HCC (aHCC) patients with ipsilateral multifocal lesions or macroscopic vascular invasion (MVI) not reach the main branch, and no distant metastasis are ideal candidates for conversion therapies based on interventional hepatic arterial infusion chemotherapy with fluorouracil, leucovorin, and oxaliplatin (FOLFOX-HAIC), which has been demonstrated to be superior in overall response rate (ORR) and higher proportion of patients who receive subsequent surgical resection^[9]^. However, the efficacy and safety of combining FOLFOX-HAIC with immunotherapy in the perioperative setting remain to be elucidated. We performed this phase II trial to evaluate the survival benefit and safety of FOLFOX-HAIC plus sintilimab, a programmed death 1 (PD-1) inhibitor, as conversion therapy for patients with locally aHCC.

## Methods

### Study design

This is a single-center, open-label, phase II trial consisting of two cohorts (https://clinicaltrials.gov/, NCT03869034). Patients were assigned to receive combination therapy of FOLFOX-HAIC plus sintilimab (cohort A, *N* = 30) or FOLFOX-HAIC monotherapy (cohort B, *N* = 10) according to their consents. The study was approved by the institutional review board of our center (B2019-001-01) on 20 February 2019 and was conducted in accordance with the Good Clinical Practice guidelines. The trial was conducted in accordance with the Declaration of Helsinki. All patients provided written informed consent. The study protocol is available in the supplementary materials. The work has been reported in line with the STROCSS criteria^[10]^.

### Patients

The study enrolled treatment-naïve adult patients (aged between 18 and 70 years) with pathologically proven HCC at the Barcelona Clinic Liver Cancer (BCLC) C stage, with at least one measurable lesion according to Response Evaluation Criteria in Solid Tumors version 1.1 (RECIST v1.1)^[11]^. Eligible patients were Child–Pugh liver function class A and Karnofsky performance status score ≥90, with adequate hematological and organ function and no contraindications for transarterial interventional therapy and PD-1 inhibitor.

Patients were required to meet the Sun Yat-sen University Cancer Center (SYSUCC) criteria for conversion therapy, defining as (1) tumor macroscopic invasion to branches of the portal vein (PV) and/or hepatic vein without main PV or inferior vena cava (IVC) invasion; (2) single or multiple tumor(s) confined to the same liver lobe; (3) no extrahepatic metastases; and (4) ECOG performance status 0–1 with Child–Pugh A liver function. In brief, all recruited patients were BCLC C stage with macrovascular invasion limited to branch vessels per SYSUCC criteria.

### Procedures

For patients in cohort A, sintilimab 200 mg was administered by intravenous infusion on day 1 of each cycle. Then, the femoral artery was punctured and chemotherapeutic agents (oxaliplatin 130 mg/m^2^, leucovorin 200 mg/m^2^, and fluorouracil 400 mg/m^2^ bolus followed by 2400 mg/m^2^ over 46 h) were infused through a percutaneous catheter inserted and placed in the tumor feeding artery identified by repeated arteriography at each course. Patients in cohort B received the same procedure and chemotherapeutic of FOLFOX-HAIC except for sintilimab. The HAIC treatments were repeated every 3 weeks until successfully oncological conversion, or disappearance of any intratumoral arterial enhancement, stenosis or occlusion of tumor feeding artery, or intolerable toxicity. Radiological assessment and multi-disciplinary consultation (MDT) were organized to decide the chance of surgery and subsequent treatment per 2 treatment cycles.

**HIGHLIGHTS**

- Patients with locally advanced hepatocellular carcinoma (aHCC) who have macroscopic vascular invasion (MVI) typically have a poor prognosis.
- Hepatic arterial infusion chemotherapy (HAIC) plus sintilimab exhibited 19.8 months of median progression-free survival and a 70% conversion-to-surgery rate in locally aHCC patients with MVI.
- Locally aHCC patients could benefit from HAIC plus sintilimab if they suffered from tumor macroscopic invasion to branches of the portal vein and/or hepatic vein.

Successfully oncological conversion was defined as tumors that achieved partial response or stable disease with shrinkage per RECIST v1.1 and a significant decrease in tumor biomarkers of ≥20%^[12]^. Patients who were assessed as technically resectable and achieved successfully oncological conversion were recommended for radical surgery. After recovering from surgery, patients in cohort A continued to receive sintilimab monotherapy per 3 weeks for a total of 16 doses, while patients in cohort B received no adjuvant therapy. Patients who were ineligible for surgery would continue to receive the study treatment for maximally eight cycles. Patients presented with radiological progressive disease (PD) or intolerable adverse events (AEs) would discontinue the study treatment and receive other treatment based on the decision of MDT. No other antitumor therapies were allowed before PD was confirmed in both cohorts, and antivirus medicine was prescribed to all patients with positive hepatitis B virus (HBV) surface antigen (HBsAg).

Radiological assessments were done with either contrast computed tomography (CT) or magnetic resonance imaging (MRI) by investigators and an independent radiologist every two cycles over the course of treatment, then every 3 months within the first 2 years and every 6 months thereafter, until PD was recorded. All subjects would be followed until death or loss to follow-up. For patients who received surgery, tissue samples were assessed for pathologic response, including the proportion of viable tumor cells, necrosis, and tumor-infiltrating lymphocytes, by two experienced pathologists. Major pathological response (MPR) was defined as the presence of 10% or fewer viable tumor cells in the tumor lesions, and pathological complete response (pCR) was defined as no viable tumor cells in the whole specimen.

### Endpoints

The primary endpoint was progression-free survival (PFS) per RECIST v1.1, defined as the duration from treatment initiation to disease progression, tumor relapse after surgery, or death from any cause, which occurred first. The core secondary endpoints included overall survival (OS, defined as the duration from treatment initiation to death from any cause), conversion rate (defined as the proportion of patients undergoing surgery of curative intent), and safety. Other efficacy outcomes included ORR, disease control rate (DCR), pathologic response, and recurrence-free survival (RFS), which was measured from the date of surgery to tumor relapse or death from any cause in the patients who received surgical resection. Safety was assessed until 100 days after the last dose of study treatments using the National Cancer Institute Common Terminology Criteria for Adverse Events (NCI-CTCAE) version 4.03.

### Statistical analysis

When this study was launched in 2019, the historical data and expected effect size for this population were unavailable. The sample size for this pilot study was arbitrarily set at 40, including two intervention arms. Cohort A included 30 patients who received FOLFOX-HAIC combined with sintilimab. Cohort B included 10 patients who received only FOLFOX-HAIC. Patients in cohort B were to be enrolled during the same period at a 3-to-1 ratio as a parallel reference, but not for statistical comparison.

**Figure 1.**
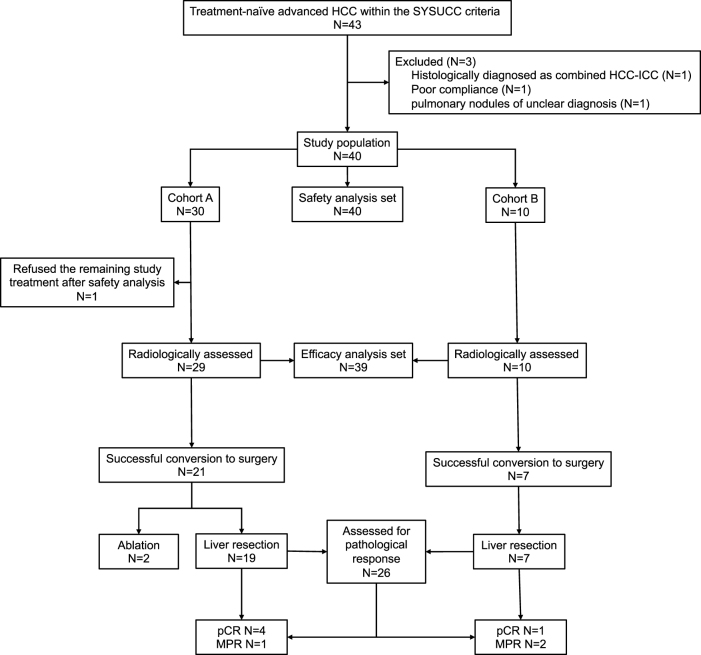
**Study flowchart and the population for analysis.** HCC, hepatocellular carcinoma; SYSUCC, Sun Yat-sen University Cancer Center; pCR, pathological complete response; MPR, major pathological response.

The analysis sets for efficacy and safety were shown in Figure 1. The Kaplan–Meier (K-M) method was used to estimate median and 95% confidence intervals (95% CIs) for PFS, OS, and RFS. Descriptive summaries were provided for all other efficacy and safety endpoints. All *P* values were two-sided, with *P* values less than 0.05 being considered statistically significant. Statistical analysis was performed using R 4.2.1 (R Foundation for Statistical Computing, Vienna, Austria).

## Results

### Study population

Between March 2019 and July 2020, 40 eligible patients were enrolled and assigned to the 2 cohorts (Fig. 1). Generally, the median age was 50 years (range, 34–70 years), 95% of the patients were male, and HBsAg was positive in 92% of the patients. The median maximal tumor diameter was 9.7 cm (range, 4.4–17.8 cm). In addition, 45% of the patients had more than one lesion (Table 1).

**Table 1:** Patient demographic and baseline characteristics.

Variables	Cohort A	Cohort B
	FOLFOX-HAIC + sintilimab (*N* = 30)	FOLFOX-HAIC (*N* = 10)
Age (range), years	51 (34–70)	49 (39–69)
Sex		
Female	2 (7%)	0 (0%)
Male	28 (93%)	10 (100%)
Etiology		
HBsAg (+)	29 (97%)	8 (80%)
Non-viral	1 (3%)	2 (20%)
α-Fetoprotein concentration		
<400 ng/mL	10 (33%)	6 (60%)
≥400 ng/mL	20 (67%)	4 (40%)
PIVKA-II		
<40 mAU/mL	1 (3%)	0 (0%)
≥40 mAU/mL	29 (97%)	10 (100%)
Number of tumors		
Single	16 (53%)	6 (60%)
Multinodular	14 (47%)	4 (40%)
Median tumor size (range), cm	9.7 (5.8–17.8)	9.7 (4.4–16.3)
Macrovascular invasion	30 (100%)	10 (100%)
Portal vein invasion^a^	26 (87%)	8 (80%)
Vp1	5 (17%)	2 (20%)
Vp2	15 (50%)	5 (50%)
Vp3	6 (20%)	1 (10%)
Hepatic vein invasion	15 (50%)	2 (20%)
Child–Pugh class A	30 (100%)	10 (100%)
KPS score ≥90	30 (100%)	10 (100%)

HAIC, hepatic arterial infusion chemotherapy; HBsAg, hepatitis B virus surface antigen; KPS, Karnofsky performance scale; PIVKA-II, protein induced vitamin K absence or antagonist-II.

^a^ The extent of portal vein tumor thrombosis (PVTT) is documented according to the Liver Cancer Study Group of Japan classification: Vp0 = no PVTT: Vp1 = segmental PV invasion; Vp2 = right anterior/posterior PV invasion; Vp3 = right/left PV invasion; and Vp4 = main trunk.

Data are presented as *N* (%) unless otherwise indicated.

### Study treatment overview

At the data cutoff date (December 2024), all patients had completed their study treatment. The median course of FOLFOX-HAIC was 2 courses (range, 2–6 courses) for both cohorts, and patients in cohort A received a median of 12 cycles of sintilimab (range, 2–16 cycles).

### Progression-free survival

**Figure 2.**
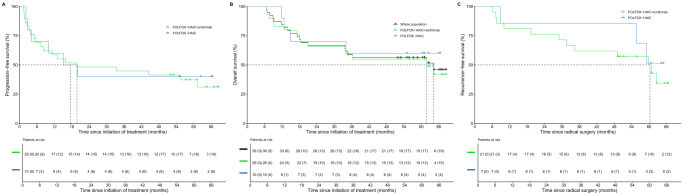
**Prognostic outcomes.** (A) K-M curves demonstrated PFS of the two cohorts. (B) K-M curves demonstrated OS of the two cohorts and the whole population. (C) K-M curves demonstrated RFS of the patients who received conversion surgery in the two cohorts. Numbers of patients at risk at the indicated time points are shown beneath the *x*-axis. K-M, Kaplan–Meier; PFS, progression-free survival; OS, overall survival; RFS, recurrence-free survival; FOLFOX-HAIC, hepatic arterial infusion chemotherapy of infusion fluorouracil, leucovorin, and oxaliplatin.

The median follow-up duration was 52.9 months (range 6.6–68.6 months). Of the 39 patients in the efficacy analysis set, 17 events in cohort A and 7 in cohort B were observed. The median PFS were 19.8 and 17.6 months [95% CI, 8.20 months-not reached (NR) and 4.13 months-NR, respectively] for cohort A and cohort B, respectively. And the 12-, 24-, and 36-month PFS rates were 59% (95% CI, 43–80%), 48% (95% CI, 33–70%), and 45% (95% CI, 30–67%) for cohort A, and 60% (95% CI, 36–100%), 40% (95% CI, 19–85%), and 30% (95% CI, 12–77%) for cohort B (Fig. 2A).

### Overall survival

The 5-year OS rate of the whole study population was 56.3% (95% CI, 43–74%), and the median OS was 61.8 months (95% CI, 34.0 months-NR) for cohort A and NR for cohort B, respectively (Fig. 2B).

### Recurrence-free survival

Among the 28 patients who underwent radical surgery, 15 (54%) experienced recurrence by the cutoff date, including 12 out of 21 patients (57%) in cohort A and 3 out of 7 patients (43%) in cohort B, respectively. The median RFS was 60.4 months (31.5 months-NR) for cohort A and NR (55.6 months-NR) for cohort B, respectively (Fig. 2C).

### Objective response rates and disease control rates

**Figure 3.**
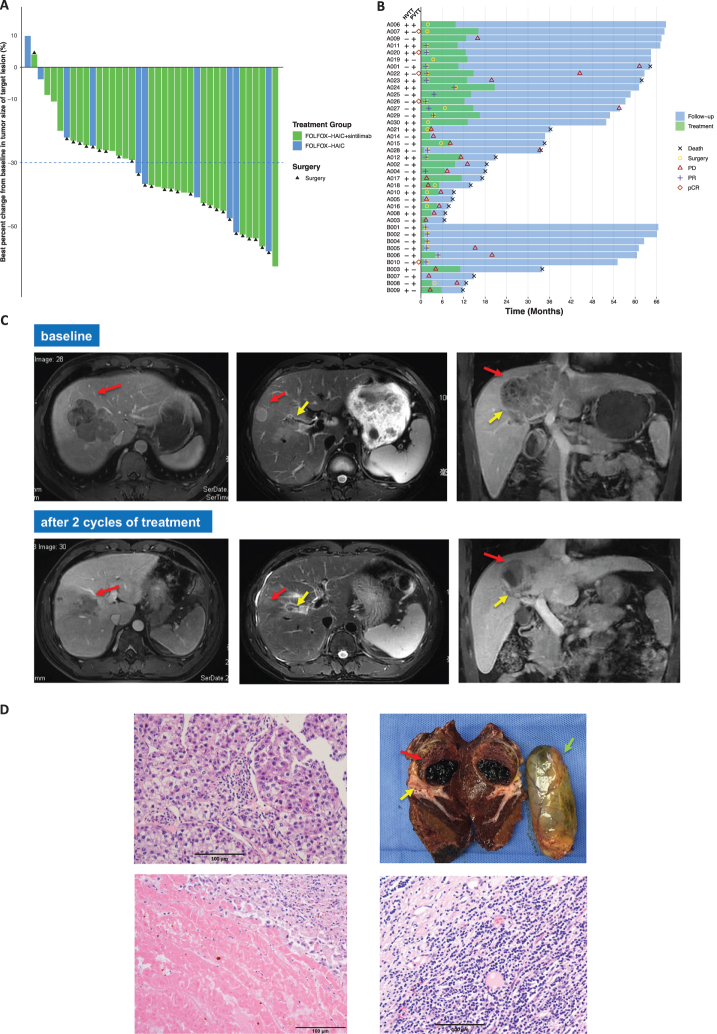
**Efficacy outcomes.** (A) Waterfall plot of the best percentage changes for the sum of target lesion diameters. The light-blue dashed lines were referenced for a 30% reduction and 20% increase in the target lesion size, respectively. Each bar in the x-axis represents individuals in the efficacy-evaluable population. (B) Duration of treatment timelines and response assessments by the RECIST v1.1 for individual HCC patients. The length of each bar represents the duration of treatment timelines (months). BOR was evaluated per the RECIST v1.1. The duration of treatment was colored coded in light green, and the duration of follow-up was in light blue. The critical events of individuals were labeled with shapes on the corresponding time points, which were illustrated in the legend. The A and B represented cohort A and cohort B. A representative case (A020) was a 34-year-old male with multiple tumors and invasion of the right branch of the portal vein. After two courses of FOLFOX-HAIC plus sintilimab, his AFP decreased from >121 000 ng/mL to 36.48 ng/mL and further dropped below 20 ng/mL post-surgery. The largest tumor shrank from 8.0 to 3.5 cm, with radiological assessment indicating PR. (C) The MRI images of the patient. Left, the primary tumor (red arrow); middle, the satellite lesion (red arrow) and tumor thrombus in the right anterior branch of portal vein (yellow arrow); right, coronal images of the primary tumor (red arrow) as well as the tumor thrombus (yellow arrow). (D) Pathological images. Top left, core biopsy before treatment revealed moderately differentiated HCC; top right, gross specimens of the resected liver tumor (red arrow) and the tumor thrombus (yellow arrow), as well as the gall bladder (green arrow) after two cycles of study treatment. The primary tumor exhibited necrosis and hemorrhage. Bottom left, extensive necrosis without any residual cancer cells in the resected tumor; bottom right, abundant tumor-infiltrating lymphocytes in the peritumoral stroma. FOLFOX-HAIC, hepatic arterial infusion chemotherapy of infusion fluorouracil, leucovorin, and oxaliplatin; HCC, hepatocellular carcinoma; BOR, best overall response; PVTT, portal vein tumor embolus; HVTT, hepatic vein tumor embolus; PD, progressive disease; PR, partial response; pCR, pathological confirmed complete response; RECIST v1.1, Response Evaluation Criteria in Solid Tumors version 1.1; MRI, magnetic resonance imaging.

The ORRs were 45% (95% CI, 26–64%) and 40% (95% CI, 14–73%) for cohort A and cohort B, respectively. The DCRs for cohort A and cohort B were 83% (95% CI, 64–94%) and 70% (95% CI, 35–92%), respectively (Table 2). Of the 39 evaluable population in efficacy analysis set, 37 (95%) achieved different extents of shrinkage of their target lesions, and 22 (16 of cohort A and 6 of cohort B) of them had a greater than 30% shrinkage of target lesion (Fig. 3A). Notably, 20 (51%) patients achieved the chance of surgery at the first time of assessment (Fig. 3B).

**Table 2:** Summary of best overall response in efficacy-evaluable HCC patients per Response Evaluation Criteria in Solid Tumors v1.1.

	Cohort A	Cohort B	Total population for efficacy analysis (*N* = 39)
	FOLFOX-HAIC + sintilimab (*N* = 29)	FOLFOX-HAIC (*N* = 10)	
Best overall response before surgery			
Complete response	0 (0%)	1 (10%)	1 (3%)
Partial response	13 (45%)	4 (40%)	17 (43%)
Stable disease	11 (38%)	2 (20%)	13 (33%)
Progressive disease	5 (17%)	3 (30%)	8 (21%)
Objective response rate *N* (%, 95% CI)	13 (45%, 26–64%)	5 (50%, 14–73%)	18 (46%, 31–61%)
Disease control rate *N* (%, 95% CI)	24 (83%, 64–94%)	7 (70%, 35–92%)	31 (79%, 67–92%)
Time to response median (95% CI); months	1.6 (1.4–2.7)	1.7 (1.8–3.5)	1.9 (1.5–7.7)
Conversion rate *N* (%, 95% CI)	21 (72%, 53–87%)	7 (70%, 35–92%)	28 (72%, 59–82%)

FOLFOX-HAIC, HCC, hepatocellular carcinoma; hepatic arterial infusion chemotherapy of infusion fluorouracil, leucovorin, and oxaliplatin; 95% CI, 95% confidence interval.

### Conversion rates

There were 21 (21/29, 72%; 95% CI, 53–87%) and 7 (7/10, 70%; 95% CI, 5–92%) patients successfully converted to radical surgery in cohort A and cohort B, respectively (Table 2). The median time from initial treatment to surgery was 1.6 months (95% CI, 1.4–2.7 months) and 1.7 months (95% CI, 1.8–3.5 months) for cohorts A and B, respectively. Notably, three patients in cohort A reported suspected lung metastasis when the target lesions shrank by over 30% and one of them underwent hepatic surgical resection after fully discussing it with the patient (Fig. 3B; A018).

### Pathologic response after resection

Of the 26 patients who received resection, 8 (31%) patients demonstrated MPR, including 5 (19%) pCR. The results of pathological evaluation were shown in Supplemental Digital Content Table S1, available at: http://links.lww.com/JS9/G631. A representative case (A020) who achieved pCR after merely 2 cycles of study treatment was shown in Figure 3C–D. The patient remains disease-free at the data cutoff with a PFS of 64.4 months.

### Safety

Any grade of treatment-related adverse events (TRAEs) occurred in all patients in both cohorts; moreover, 28 patients (93%) and 9 patients (90%) in cohort A and cohort B, occurred greater than grade 3 TRAEs, respectively, with no treatment-related death. The most frequent TRAEs were transitory decreased albumin, aspartate aminotransferase, and elevated alanine aminotransferase, which mostly occurred after HAIC and recovered soon without sequelae. No AEs of preoperative treatment prevented any patients from undergoing surgery. Only one patient of cohort A experienced grade 4 postoperative hepatic failure, which was judged not to be immune-related based on histological examination. This patient permanently discontinued the study treatment due to this severe AE. No AE-related death occurred. Details of TRAEs were shown in Table 3.

**Table 3:** Treatment-related adverse events (TRAEs).

	Cohort A					Cohort B				
	FOLFOX-HAIC + sintilimab (*N* = 30)					FOLFOX-HAIC (*N* = 10)				
TRAEs	Any grade	Grade 1	Grade 2	Grade 3	Grade 4	Any grade	Grade 1	Grade 2	Grade 3	Grade 4
Reduced WBC count	8 (27%)	3 (10%)	5 (17%)	0	0	2 (20%)	1 (10%)	1 (10%)	0	0
Anemia	21 (70%)	13 (43%)	4 (13%)	4 (13%)	0	7 (70%)	4 (40%)	2 (20%)	1 (10%)	0
Reduced platelet count	13 (43%)	4 (13%)	4 (13%)	5 (17%)	0	3 (30%)	3 (30%)	0	0	0
ALT elevation	24 (80%)	6 (20%)	6 (20%)	11 (37%)	1 (3%)	10 (100%)	2 (20%)	0	8 (80%)	0
AST elevation	26 (87%)	4 (13%)	6 (20%)	15 (50%)	1 (3%)	10 (100%)	1 (10%)	0	7 (70%)	2 (20%)
Decreased albumin	27 (90%)	21 (70%)	6 (20%)	0	0	10 (100%)	8 (80%)	1 (10%)	1 (10%)	0
Increased TBIL	17 (57%)	8 (27%)	7 (23%)	1 (3%)	1 (3%)	6 (60%)	3 (30%)	3 (30%)	0	0
Abdominal pain	18 (60%)	11 (37%)	6 (20%)	1 (3%)	0	3 (30%)	0	3 (30%)	0	0
Diarrhea	3 (10%)	2 (7%)	1 (3%)	0	0	0	0	0	0	0
Arrhythmia	2 (7%)	2 (7%)	0	0	0	0	0	0	0	0
Vomiting	10 (33%)	4 (13%)	4 (13%)	2 (7%)	0	3 (30%)	1 (10%)	2 (20%)	0	0
Dry mouth	1 (3%)	1 (3%)	0	0	0	0	0	0	0	0
Rash	7 (23%)	5 (17%)	2 (7%)	0	0	0	0	0	0	0
Pruritus	3 (10%)	3 (10%)	0	0	0	0	0	0	0	0
Pyrexia	18 (60%)	13 (43%)	5 (17%)	0	0	6 (60%)	6 (60%)	0	0	0
Acroparesthesia	2 (7%)	2 (7%)	0	0	0	0	0	0	0	0
Hypothyroidism	2 (7%)	2 (7%)	0	0	0	0	0	0	0	0
Adrenal insufficiency	1 (3%)	1(3%)	2 (7%)	0	0	0	0	0	0	0
Hepatic failure^a^	1 (3%)	0	0	0	1 (3%)	0	0	0	0	0

ALT, alanine transaminase; AST, aspartate aminotransferase; FOLFOX-HAIC, hepatic arterial infusion chemotherapyof infusion fluorouracil, leucovorin, and oxaliplatin; TBIL, total bilirubin; TRAEs, treatment-related adverse events; WBC, white blood cell.

^a^ Occurred post conversion surgery.

## Discussion

HCC patients with MVI suffered from a poor prognosis if they underwent resection without perioperative therapies. Even for HCC patients with Vp1–2 MVI, the 1-year OS rate after resection was less than 50%. Additional treatment was urgently required to improve the long-term prognosis of these patients^[13,14]^. FOLFOX-HAIC-based regimen are widely used as conversion therapy for HCC with MVI in China, intending to bring a chance for surgical resection. However, the survival benefit of the conversion-to-surgery strategy for locally aHCC remains unclear. This study used perioperative PD-1 therapy in HCC patients with MVI who underwent FOLFOX-HAIC conversion, utilizing PFS as a measure of long-term survival. With a quite long follow-up duration of 52.9 months, the patients achieved a median PFS of 19.6 months, the estimated 5-year OS rate of 56.3% with a manageable safety profile.

One reason for the impressive long-term prognosis of the present study could be attributed to the “SYSUCC criteria” which refined the study population. As hepatectomy failed to provide survival benefits for HCC patients with tumor thrombosis extending to the main PV or IVC in a previous study, the SYSUCC criteria exclude these patients, besides patients with extrahepatic metastases^[15]^. Furthermore, excluding patients with spread lesions in both lobes of the liver provided reassurance of less surgical injury and enough liver function preservation, which are quite important for long-term survival. On the other hand, FOLFOX-HAIC has priority in treating HCC larger than 7 cm, and the median diameter of tumors of the study population was 9.7 cm, which just falls into the advantageous population of study treatment^[9]^. Moreover, in the EMERALD-1 subgroup analysis, a phase III trial evaluating durvalumab plus bevacizumab in combination with TACE for unresectable HCC, TACE with durvalumab plus placebo did not significantly improve PFS compared with TACE plus placebo [median 10.0 months (95% CI, 9.0–12.7) vs. 8.2 months (95% CI, 6.9–11.1); hazard ratio (HR), 0.94 (95% CI, 0.75–1.19); *P* = 0.64]^[16]^. In the present study, similarly, locoregional FOLFOX-HAIC plus sintilimab did not show improved PFS versus FOLFOX-HAIC alone consistently.

Conversion strategies for patients with locally aHCC are still under active exploration as drugs and techniques continue to advance. Recently, Ho *et al* and colleagues reported that the neoadjuvant cabozantinib and nivolumab convert locally aHCC patients with an ORR of 7% and DCR of 100%, respectively^[17]^. In China, the locoregional-based combination strategies are the mainstream for conversion purposes; the TACE combined with lenvatinib demonstrated 7.5 times higher rates of conversion than lenvatinib alone (15 vs. 2%) for locally aHCC^[18]^. Furthermore, the triplet combination of FOLFOX-HAIC, lenvatinib, and toripalimab exhibited a conversion rate of 22% in nonselected locally aHCC^[19]^. The present trial was designed to assess the efficacy and safety of FOLFOX-HAIC, either alone or in combination with a PD-1 inhibitor, as a conversion therapy. Theoretically, sintilimab may modulate the immune microenvironment to influence apoptosis-related pathways, thereby synergizing with and enhancing the therapeutic efficacy of FOLFOX-HAIC^[20]^. Notably, the grade 3–4 TRAEs of the present study were obviously lower than those of the triple combination (13.3 vs. 38.9%)^[19]^. In this study, the median treatment cycle of HAIC before conversion was identical between the two cohorts, hinting that adding sintilimab did not shorten the time to response. However, considering the small sample size and uncontrolled design of this study, these findings should be interpreted cautiously. Overall, FOLFOX-HAIC plus a PD-1 inhibitor could be considered an optimal conversion strategy, offering impressive efficacy and good tolerance for selected patients with locally aHCC.

The current study has several limitations. First, the 3:1 allocation between cohorts A and B and the absence of prespecified between-group hypothesis testing limit the strength of any comparative conclusions. Second, the study population consisted solely of Chinese patients with predominantly HBV-related, large HCC treated at a single center, which restricts the generalizability of the findings. These raise the need to explore the efficacy and safety of FOLFOX-HAIC in patients with other etiologies, as well as conducting randomized controlled trials comparing FOLFOX-HAIC plus immune therapy with target medicine plus immune therapy as conversion therapy in locally aHCC patients within SYSUCC criteria in the future.

## Conclusions

Hepatic arterial infusion chemotherapy plus sintilimab exhibited impressive efficacy and manageable safety as conversion therapy for locally aHCC, and the conversion-to-surgery could obtain long-term survival benefit in selective patients with locally aHCC.

## Data Availability

Datasets generated during and/or analysed during the current study are publicly available, available upon reasonable request.

## Research registration unique identifying number (UIN)

Clinicaltrials.gov, NCT03869034.
